# Genome assembly of the chemosynthetic endosymbiont of the hydrothermal vent snail *Alviniconcha adamantis* from the Mariana Arc

**DOI:** 10.1093/g3journal/jkac220

**Published:** 2022-08-23

**Authors:** Corinna Breusing, Nathan Hagen Klobusnik, Michelle A Hauer, Roxanne A Beinart

**Affiliations:** Graduate School of Oceanography, University of Rhode Island, Narragansett, RI 02882, USA; Texas A&M University at Galveston, Galveston, TX 77554, USA; Graduate School of Oceanography, University of Rhode Island, Narragansett, RI 02882, USA; Graduate School of Oceanography, University of Rhode Island, Narragansett, RI 02882, USA

**Keywords:** chemosynthetic symbiosis, hydrothermal vents, *Alviniconcha adamantis*, Mariana Arc

## Abstract

Chemosynthetic animal-microbe symbioses sustain hydrothermal vent communities in the global deep sea. In the Indo-Pacific Ocean, hydrothermal ecosystems are often dominated by gastropod species of the genus *Alviniconcha*, which live in association with chemosynthetic Gammaproteobacteria or Campylobacteria. While the symbiont genomes of most extant *Alviniconcha* species have been sequenced, no genome information is currently available for the gammaproteobacterial endosymbiont of *Alviniconcha adamantis—*a comparatively shallow living species that is thought to be the ancestor to all other present *Alviniconcha* lineages. Here, we report the first genome sequence for the symbiont of *A. adamantis* from the Chamorro Seamount at the Mariana Arc. Our phylogenomic analyses show that the *A. adamantis* symbiont is most closely related to Chromatiaceae endosymbionts of the hydrothermal vent snails *Alviniconcha strummeri* and *Chrysomallon squamiferum*, but represents a distinct bacterial species or possibly genus. Overall, the functional capacity of the *A. adamantis* symbiont appeared to be similar to other chemosynthetic Gammaproteobacteria, though several flagella and chemotaxis genes were detected, which are absent in other gammaproteobacterial *Alviniconcha* symbionts. These differences might suggest potential contrasts in symbiont transmission dynamics, host recognition, or nutrient transfer. Furthermore, an abundance of genes for ammonia transport and urea usage could indicate adaptations to the oligotrophic waters of the Mariana region, possibly via recycling of host- and environment-derived nitrogenous waste products. This genome assembly adds to the growing genomic resources for chemosynthetic bacteria from hydrothermal vents and will be valuable for future comparative genomic analyses assessing gene content evolution in relation to environment and symbiotic lifestyles.

## Introduction

While most areas of the deep sea depend on sinking organic particles originating from photosynthetic primary production at the ocean’s surface, ecosystems around deep-sea hydrothermal vents are fueled by the biochemical processes carried out by chemosynthetic microbes. These organisms are typically chemolitho- or chemoorganotrophic Gammaproteobacteria or Campylobacteria that oxidize reduced hydrothermal fluid compounds, such as sulfide, hydrogen, or methane, to generate energy for carbon fixation (Sogin *et al.* 2020, 2021). Many chemosynthetic microbes are known to form symbiotic relationships with vent-associated invertebrate animals, thereby supplying these hosts with the bulk of their nutritional requirements and leading to the high animal biomass that is characteristic of hydrothermal vent communities (Dubilier *et al.* 2008; Sogin *et al.* 2020, 2021).

A diversity of chemosynthetic symbioses has been discovered and described, including that of the hydrothermal vent snail *Alviniconcha* (Suzuki *et al.* 2006; Johnson *et al.* 2015; Breusing, Johnson *et al.* 2020; Breusing, Castel *et al.* 2022), a genus of endangered foundation fauna found at hydrothermal vents across the Western Pacific and Indian oceans (https://www.iucnredlist.org; last accessed: August 27, 2022). Most *Alviniconcha* species foster symbiotic associations with chemosynthetic Gammaproteobacteria that are assumed to be environmentally acquired and reside intracellularly within the snail’s gill tissue (Suzuki *et al.* 2006; Breusing, Castel *et al.* 2022). Previous genome reports and physiological experiments have shown that *Alviniconcha* symbionts primarily use reduced sulfur compounds and, in some cases, hydrogen as energy sources for their chemosynthetic metabolism (Beinart *et al.* 2015; Miyazaki *et al.* 2020; Breusing, Mitchell *et al.* 2020), while likely additionally synthesizing essential amino acids for their hosts (Beinart *et al.* 2019).

With the exception of *Alviniconcha adamantis*, the dominant endosymbiont genomes of all known *Alviniconcha* species have been sequenced (Beinart *et al.* 2019; Trembath-Reichert *et al.* 2019; Yang *et al.* 2020; Breusing, Genetti *et al.* 2022; Hauer *et al.* 2022). *Alviniconcha adamantis* is endemic to the Mariana Arc, where it inhabits relatively shallow seamounts in contrast to its deeper living congeners. Due to its basal (though uncertain) phylogenetic position, recent studies have hypothesized that *A. adamantis* might be the ancestor to all other extant *Alviniconcha* species, supporting an evolutionary transition from shallow to deep water vent sites (Breusing, Johnson *et al.* 2020). How the distinct ecological niche of *A. adamantis* might have shaped gene content and functional potential of its gammaproteobacterial symbiont is currently unknown. Understanding symbiont metabolic capacity can help us infer fundamental characteristics of hydrothermal vent ecology and evolution, giving us insights into how chemosynthetic microbes interact with and adapt to their biogeochemical environment.

In this study, we sequenced a draft genome of the endosymbiont of *A. adamantis* from the Mariana Arc. Using comparative genomic and phylogenomic analyses, we determined its phylogenetic placement with respect to other chemosynthetic Gammaproteobacteria and compared its metabolic potential with that of related vent-associated symbionts.

## Materials and methods

### Sample collection, nucleic acid extraction, and sequencing

**Fig. 1.**
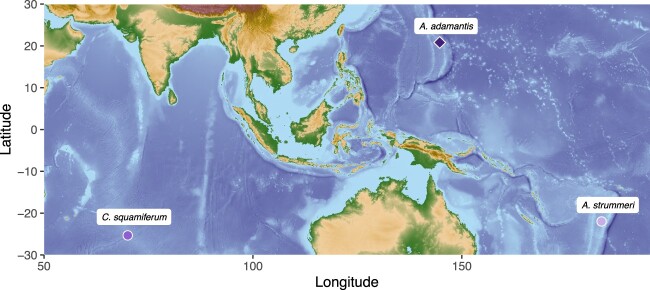
Sampling location of *Alviniconcha adamantis* in the Mariana Arc, from which the symbiont genome reported here was isolated. Habitats of other host species with closely related symbionts are shown, *A. strummeri* in the Lau Basin and *Chrysomallon squamiferum* on the Central Indian Ridge. The map was produced with the marmap package (Pante and Simon-Bouhet, 2013) in R.

The samples of *A. adamantis* were collected from Chamorro Seamount (20°49'12.0″N 144°42'36.0″E, 920 m) at the Mariana Arc in 2016 during R/V *Falkor* cruise FK161129 with the ROV *SuBastian* (Fig. 1). Symbiont-bearing gill tissue was excised and preserved in RNALater (Thermo Fisher Scientific, Inc., Waltham, MA, USA) at −80°C until further analysis. DNA was extracted with the Zymo Quick DNA 96 Plus and ZR-96 Clean-up kits (Zymo Research, Inc., Irvine, CA, USA) and submitted for Illumina 150 bp paired-end library preparation and sequencing at Novogene Corporation (Beijing, China). Raw reads were trimmed with Trimmomatic v0.36 (Bolger *et al.* 2014) with the following parameters, ILLUMINACLIP: Illumina.fa: 2:30:10 SLIDINGWINDOW: 4:20 LEADING: 5 TRAILING: 5 MINLEN: 75, and then filtered for sequence contaminants through mapping against the human (GRCh38) and PhiX reference genomes. High molecular weight DNA for additional Nanopore sequencing runs was extracted with Qiagen Genomic Tips (Qiagen, Inc., Hilden, Germany) and enriched for fragments >25 kb with the Circulomics Short-Read Eliminator kit (PacBio, Menlo Park, CA, USA). Nanopore libraries were constructed with the SQK-LSK109 ligation kit and sequenced on 2 separate flow cells on a MinION device (Oxford Nanopore Technologies, Oxford, UK). Basecalling of the Nanopore reads was done locally with MinKNOW v4.2.8 in high accuracy mode and adapters were clipped with Porechop v0.2.4 (https://github.com/rrwick/Porechop; last accessed: August 27, 2022).

### Genome assembly, binning, and annotation

Hybrid assemblies of Illumina and Nanopore reads were constructed with metaSPAdes v3.13.1 (Nurk *et al.* 2017) using kmers from 21 to 121 in 10-step increments, manually binned with gbtools (Seah and Gruber-Vodicka 2015) and then reassembled with SPAdes (Bankevich *et al.* 2012) in careful mode with automatic coverage cutoff using only symbiont reads that mapped against the metaSPAdes bin (Supplementary Table 1). The SPAdes assembly was scaffolded and gapfilled with SSPACE v3.0 (Boetzer *et al.* 2011) and GapFiller v1.10 (Boetzer and Pirovano 2012), respectively. Scaffolds smaller than 200 bp were excluded. The final assembly was polished with Pilon v1.22 (Walker *et al.* 2014) with the “–fix-all –changes” options and assessed for completeness and contamination with checkM v1.0.13 (Parks *et al.* 2015) based on 280 Gammaproteobacteria-specific marker genes. General assembly statistics were quantified with QUAST v5.0.0 (Gurevich *et al.* 2013). Protein-coding genes were predicted with Prodigal v2.6.3 (Hyatt *et al.* 2010) and functionally annotated with the KEGG (Kanehisa *et al.* 2016) and COG (Galperin *et al.* 2015) databases in Anvi’o v7.1 (Eren *et al.* 2015) using Blastp (Camacho *et al.* 2009) for protein sequence comparisons. Ribosomal and transfer RNAs were inferred with Barrnap v0.9 (https://github.com/tseemann/barrnap; last accessed: August 27, 2022) and tRNAscan-SE v2.0.9 (Chan *et al.* 2021), respectively. Putative hydrogenase genes were classified with HydDB (Søndergaard *et al.* 2016). Taxonomic assignment was done with GTDB-Tk v1.5.0 (Chaumeil *et al.* 2019). To evaluate the diversity of the intrahost symbiont population we called single nucleotide polymorphisms, insertion–deletions, and other variant types with FreeBayes v1.3.6 (Garrison and Marth 2012) as in Breusing, Genetti *et al.* (2022). In addition, low-frequency variants were identified through LoFreq v2.1.5 (Wilm *et al.* 2012) with default filters for coverage and strand bias, a minimum mapping quality of 30 and a minimum base quality of 20.

### Comparative genomics and phylogenomics

A phylogeny of the *A. adamantis* symbiont and representatives of other chemosynthetic Gammaproteobacteria (Supplementary Table 2) was constructed with IQ-TREE v2.0.6 (Minh *et al.* 2020) based on an amino acid alignment of concatenated single-copy core genes in the Anvi’o “Bacteria_71” collection (Eren *et al.* 2015). Phylogenomic trees were inferred from 5 independent runs based on a gene-wise best-fit partition model identified with ModelFinder using the relaxed hierarchical clustering method (Lanfear *et al.* 2014). Branch support was calculated via ultrafast bootstrapping and Shimodaira–Hasegawa-like approximate likelihood ratio tests, resampling partitions, and sites within resampled partitions 1,000 times. Bootstrap trees were optimized through a hill-climbing nearest neighbor interchange search to minimize the effect of model violations. The free-living SUP05 bacterium *Ca.* Pseudothioglobus singularis was used as outgroup for tree rooting. The best maximum likelihood tree was displayed and polished with FigTree v1.4.4 (http://tree.bio.ed.ac.uk/software/figtree/; last accessed: August 27, 2022). Gene content differences among the *A. adamantis* symbiont and related Gammaproteobacteria were assessed in Anvi’o by determining the presence and completeness of metabolic pathways via the “anvi-run-kegg-kofams” and “anvi-estimate-metabolism” programs. Modules were considered as complete when at least 75% of participating genes were found. Core and unique protein-coding genes between the *A. adamantis* symbiont and closest bacterial relatives were evaluated through the Anvi’o pangenomics workflow. Principal coordinate plots and heatmaps were produced in R v4.1.2 with the ggplot2, ComplexHeatmap, and circlize packages (Gu *et al.* 2014, 2016; Wickham 2016; R Core Team 2021) and polished in Inkscape v1.0.0b1 (https://inkscape.org; last accessed: August 27, 2022).

## Results and discussion

### Overview of the genome assembly

The *A. adamantis* symbiont draft genome consists of 427 scaffolds comprising an approximate total size of 3.3 Mb, an N50 value of 16,689 bp, and a GC content of 62.04%, with an average coverage of 931× (Table 1). Functional annotation analyses predicted 3,821 protein-coding genes, 2 rRNAs and 45 tRNAs, with 833 (21.54%) genes having no designated function (Table 1, Supplementary Table 3). About 11.63% of the genome consisted of intergenic regions. Based on Gammaproteobacteria-specific marker genes, the genome assembly is 98.88% complete with 2.06% contamination and 16.67% strain heterogeneity (Table 1). Read mapping against the *A. adamantis* symbiont genome recovered 198 variant sites based on FreeBayes but 24,332 variant sites based on LoFreq, which translates into a variant density of 7.44 variants/kbp. Given that LoFreq is optimized for detecting low-frequency variants, the discrepancy between the 2 programs suggests that the symbiont population within *A. adamantis* individuals likely consists of one dominant strain (in agreement with Breusing, Castel *et al.* 2022) as well as several low abundance strains that are only detectable with more sensitive methods.

**Table 1.** Assembly statistics for the *Alviniconcha adamantis* endosymbiont genome.

Assembly metric	
Genome size (bp)	3,268,514
Number of scaffolds	427
Longest scaffold (bp)	90,954
Scaffold N50	16,689
Scaffold L50	61
GC (%)	62.04
Ns per 100 kbp	4.04
Average coverage (X)	931
Number of coding sequences	3,821
Number of annotated CDS	2,988
Number of hypothetical CDS	833
Number of rRNAs	2
Number of tRNAs	45
Completeness (%)	98.88
Contamination (%)	2.06
Strain heterogeneity (%)	16.67

### Comparative genomics and phylogenomics

**Fig. 2.**
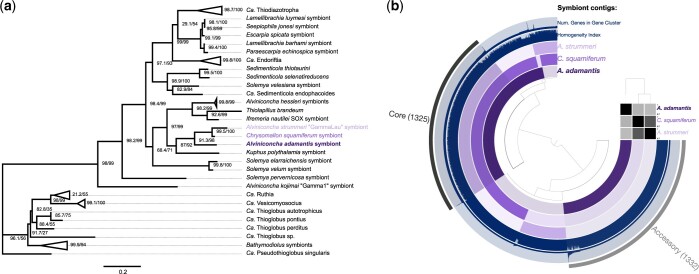
a) Representative phylogeny of chemosynthetic Gammaproteobacteria, for which whole-genome sequences were available (Supplementary Table 2). The *A. adamantis* symbiont forms a sister clade to the Chromatiaceae symbionts of *A. strummeri* and *C. squamiferum* despite the vast geographic distances among the habitats of these species. Numbers on nodes indicate support values from ultrafast bootstrapping and Shimodaira–Hasegawa-like approximate likelihood ratio tests. b) Pangenome of the *A. adamantis*, *A. strummeri*, and *C. squamiferum* symbionts. Symbiont contigs are shown as purple layers, while number of genes and combined homogeneity indices of gene clusters are shown as blue layers. The homogeneity index is a measure of amino acid sequence similarity within computed gene clusters, with higher values indicating more homogeneous clusters. The 3 symbionts share 1,325 core protein-coding gene clusters (containing 4,167 genes), while approximately the same amount of gene clusters is exclusive to the *A. adamantis* symbiont in agreement with the genomic and phylogenetic divergence among symbiont species. The matrix on the right shows average nucleotide identities among symbiont genomes from 70% to 100%, with darker grey tones indicating higher identities.

**Fig. 3.**
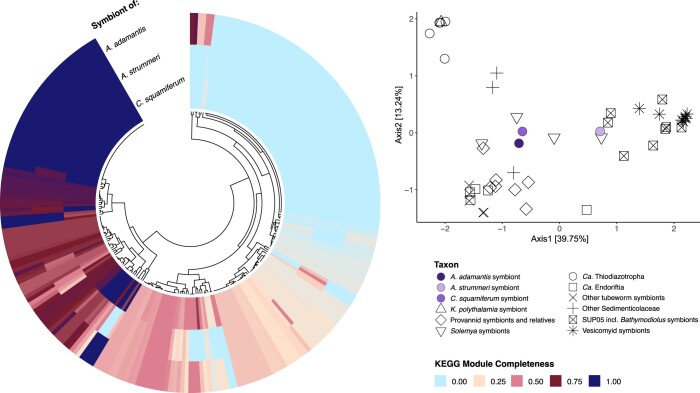
Completeness of KEGG metabolic pathways in the *A. adamantis* symbiont compared to its closest bacterial relatives (left) and functional similarity to other chemosynthetic Gammaproteobacteria (right). In contrast to phylogenetic proximity, the *A. adamantis* and *C. squamiferum* symbionts are more similar to each other in terms of functional potential than either of these species to the *A. strummeri* symbiont.

Phylogenomic analyses and taxonomic assignment indicated that the *A. adamantis* symbiont represents a sister taxon to the Chromatiaceae endosymbionts of the hydrothermal vent snails *Chrysomallon squamiferum* (from the Indian Ocean) and *Alviniconcha strummeri* (“GammaLau,” from the Lau Basin; Fig. 2, Supplementary Fig. 1), despite the fact that these symbionts and their hosts inhabit distant biogeographic provinces (Fig. 1). The *A. adamantis* symbiont shared on average 76.75% and 77.88% nucleotide identity with the *A. strummeri* and *C. squamiferum* symbionts, respectively, whereas the latter 2 taxa were less divergent, comprising an average nucleotide identity of 89.02%. The present genome similarities indicate that all 3 symbionts are representatives of distinct bacterial species (Konstantinidis and Tiedje 2005), with the *A. adamantis* symbiont possibly representing a different genus. All symbionts shared 1,325 core protein-coding gene clusters, while the *A. adamantis* symbiont contained approximately the same number of accessory gene clusters (1,332; Fig. 2, Supplementary Table 3), in accordance with the observed genomic divergence. Core genes were mostly associated with translation, energy production, and amino acid, cofactor, and cell wall metabolism, whereas accessory genes were predominantly involved in signal transduction, replication, mobilome, and defense mechanisms or had unknown functions (Supplementary Table 3). Interestingly, the phylogenetic affiliations among these taxa were not exactly mirrored in representations of functional potential, given that the *A. adamantis* and *C. squamiferum* symbionts were more similar in metabolic pathways than either of these species to the *A. strummeri* symbiont (Fig. 3, Supplementary Fig. 2). Overall, the *A. adamantis* and *C. squamiferum* symbionts exhibited functional proximity (i.e. overlap in gene content and metabolic pathways) to other provannid snail, tubeworm, and *Solemya* clam symbionts, while the *A. strummeri* symbiont showed higher affinity to bacteria of the SUP05 group (Fig. 3, Supplementary Fig. 2).

### Chemoautotrophic and heterotrophic metabolism

Both hydrogen sulfide and thiosulfate oxidation pathways were detected within the *A. adamantis* symbiont genome (Supplementary Tables 3 and 4). Oxidation of hydrogen sulfide is likely facilitated through type I and type VI sulfide: quinone oxidoreductases (*sqr*) and a flavocytochrome c-sulfide dehydrogenase (*fccAB*), which are hypothesized to be used for growth in habitats with variable sulfide concentrations (Han and Perner 2016; Beinart *et al.* 2019; Breusing, Mitchell *et al.* 2020). Typical for chemosynthetic Gammaproteobacteria (Nakagawa and Takai 2008; Gregersen *et al.* 2011), the thiosulfate-oxidizing Sox multienzyme complex (*soxXYZABC*) without a complete *soxCD* subunit was encoded, which likely promotes oxidation of sulfur compounds to elemental sulfur as energy storage in the periplasm (Grimm *et al.* 2008; Ghosh and Dam 2009). Likewise, we observed genes for the reverse dissimilatory sulfite reductase associated pathway, which catalyzes the oxidation of sulfide to sulfate via sulfite and adenylylphosphosulfate (Nakagawa and Takai 2008) and is characteristic for gammaproteobacterial sulfur-oxidizers (Gregersen *et al.* 2011). An alternative pathway for sulfite metabolization might be performed by sulfite dehydrogenase (*soeABC*).

Apart from potential for sulfur oxidation, the *A. adamantis* symbiont genome showed capacity for the usage of hydrogen as electron donor for chemosynthesis (Supplementary Table 3). We found evidence for the presence of 2 uptake Ni/Fe hydrogenases, an O_2_-tolerant hydrogenase of type 1d (gene caller ID: 3368) and an O_2_-sensitive hydrogenase of type 1e (gene caller ID: 165, 166), which are likely employed for growth under aerobic and anaerobic conditions, respectively. The expression and formation of these primary hydrogenases might be regulated by a sensory Group 2b Ni/Fe hydrogenase (gene caller ID: 3354).

As in other chemosynthetic Gammaproteobacteria (Hügler and Sievert 2011), the energy generated through hydrogen or sulfur oxidation is likely transferred to Form II RuBisCO (*cbbM*) for carbon assimilation via the Calvin–Benson–Bassham cycle, which was the only complete carbon fixation pathway found in the *A. adamantis* symbiont genome (Supplementary Tables 3 and 4). Similar to what has been reported from other *Alviniconcha* symbionts, there is evidence that the *A. adamantis* symbiont has the potential for heterotrophic metabolism. We found several transporters for the uptake of 4 carbon compounds (TRAP transport system), sugars (phosphotransferase system), lipids, amino acids, and urea in the genome of the *A. adamantis* symbiont. In addition, genes for the utilization of glycolate (glycolate oxidase), urea (urease), glycogen (glycogen phosphorylase), and formate (formate hydrogenlyase) were observed.

### Respiration

The *A. adamantis* symbiont genome encodes pathways for both aerobic and anaerobic respiration. A full set of genes of the aerobic respiratory chain was detected, including NADH-quinone oxidoreductase, succinate dehydrogenase, cytochrome bc1 complex, cytochrome cbb3-type oxidase, and an F-type ATPase (Supplementary Tables 3 and 4). In addition, subunits I, II, and X of a terminal cytochrome bd-I ubiquinol oxidase were found, which is thought to be used for aerobic respiration under microaerophilic conditions (Borisov *et al.* 2011; Beinart *et al.* 2019). The symbiont’s capacity to express different respiratory enzymes might be an adaptation to deal with fluctuating oxygen concentrations at hydrothermal vents and to remedy interference with host respiration (Beinart *et al.* 2019). Under complete anoxia, the *A. adamantis* symbiont appears to be able to switch to multiple electron acceptors other than oxygen. For example, nitrate respiration is likely supported by the presence of complete pathways for denitrification as well as dissimilatory nitrate reduction (Supplementary Tables 3 and 4). Furthermore, respiration of hydrogen and dimethyl sulfoxide seems possible through genes coding for formate hydrogenlyase and anaerobic dimethyl sulfoxide reductase.

### Nitrogen assimilation

The *A. adamantis* symbiont appears to be able to use multiple nitrogen sources for the incorporation of nitrogen into biomass. For example, we detected several genes for ammonia transporters and urease in the *A. adamantis* symbiont genome (Supplementary Table 3), which should allow direct uptake of ammonia from the environment or host and disintegration of urea into 2 ammonia molecules. Ammonia would subsequently be available for conversion into glutamine by glutamine synthetase and further incorporation into glutamate by NADPH-dependent glutamate synthase (GOGAT). Interestingly, the KEGG/COG annotation pipeline failed to recover genes for assimilatory nitrate reductase (*nasA*), which is present in other provannid symbionts (Beinart *et al.* 2019). This finding is likely an artifact of the annotation database or gene prediction program, as further searches via RAST-Tk (Brettin *et al.* 2015) indicated the presence of *nasA* in the genome of the *A. adamantis* symbiont. Nevertheless, given the oligotrophic nature of the Mariana region (Morel *et al.* 2010), the abundance of genes for ammonia transport and urea catabolism in the genome of the *A. adamantis* symbiont could suggest scavenging of host and environmental waste products in adaptation to limited nutrient availability at the Chamorro Seamount.

### Amino acid and cofactor biosynthesis

In addition to the synthesis of glutamine and glutamate, the *A. adamantis* symbiont has the potential for the generation of 13 other amino acids, including the essential amino acids histidine, isoleucine, leucine, lysine, methionine, threonine, tryptophan, and valine, which are critical for host nutrition (Supplementary Table 4). Pathways for the biosynthesis of cysteine, glycine, phenylalanine, serine, and tyrosine appeared incomplete, which might suggest reliance of the symbiont on environmental provisioning of these amino acids or could be indicative of artifacts in the assembly or functional annotations. For example, the terminal enzyme for serine biosynthesis, phosphoserine phosphatase (*serB*), was missing from the KEGG pathway predictions, but was present in the COG annotations. This could imply that the *A. adamantis* specific gene is too divergent from reference sequences in the KEGG database to be correctly annotated and that this symbiont is actually able to synthesize serine.

Apart from essential amino acid biosynthesis, pathways for the generation of diverse enzyme cofactors were observed in the *A. adamantis* symbiont genome. Based on KEGG metabolic reconstructions, the *A. adamantis* symbiont has the potential to de novo synthesize NAD, heme, siroheme, ubiquinone, molybdenum, lipoic acid and the vitamins biotin, thiamine, folate, and riboflavin (Supplementary Table 4). By contrast, conventional pathways for the biosynthesis of cobalamin, pantothenate, pyridoxal-5′ phosphate, ascorbate, and phylloquinone appeared incomplete, but might in some cases be substituted by alternative routes. For example, the lack of 2-dehydropantoate-2-reductase for the conversion of 2-dehydropantoate to (R)-pantoate might be compensated by ketol-acid reductoisomerase (*ilvC*) (Merkamm *et al.* 2003), thereby allowing autonomous generation of pantothenate and coenzyme A. In the absence of complete biosynthetic pathways, the respective cofactors will have to be acquired from an environmental source, given that several vitamin-dependent enzymes, such as cobalamin-dependent methionine synthase (*metH*) and pyridoxal-5′ phosphate-dependent cysteine-S-conjugate beta-lyase, were encoded in the *A. adamantis* symbiont genome.

### Host–symbiont interactions

Aside from chemosynthesis genes, the genome of the *A. adamantis* symbiont encodes multiple loci that are likely relevant for interactions with its host, including genes for flagella (*motAB*, *flgABC*, *flgJKLMN*, *flgZ*, *fliA, fliCDEFGHIJKLNMOPQRST*), pili (*pilABC*, *pilEFGHIJ*, *pilMNOPQ*, *pilSTUVW*, *pilZ*, *fimT*, *fimV*, *cpaBC*, *cpaF*, *tadBCD*, *tadG*), chemotaxis (*MCP*, *cheAB*, *cheD*, *cheR*, *cheVW*, *cheYZ*), toxin-antitoxin and 2-component systems (e.g. *fitAB*, *higAB*, *vapBC*, *algRZ*) as well as outer membrane porins (*ompA-F*; Supplementary Table 3). The discovery of flagella genes in the *A. adamantis* symbiont genome is surprising as these genes are typically abundant in campylobacterial, but not gammaproteobacterial *Alviniconcha* symbiont genomes (Beinart *et al.* 2019), though are observed in some other symbiotic Gammaproteobacteria, including those of tubeworms and mussels (Robidart *et al.* 2008; Egas *et al.* 2012; Gardebrecht *et al.* 2012; De Oliveira *et al.* 2022). The presence of flagella-encoding loci could suggest that the biology of the *A. adamantis* symbiosis is markedly different from other gammaproteobacterial associations in *Alviniconcha* and has closer resemblance to Campylobacteria-dominated systems, where flagella have been implicated in host specificity, nutrient transfer and/or continuous symbiont transmission (Sanders *et al.* 2013). Host specificity might further be promoted by outer membrane porins, which have been shown to play a role in host recognition in both terrestrial and aquatic symbioses (Weiss *et al.* 2008; Nyholm *et al.* 2009; Zvi-Kedem *et al.* 2021). Host colonization and subsequent maintenance of the intrahost symbiont population involves a delicate interplay between host and symbiont molecular factors. Many of the detected toxin-antitoxin and 2-component systems are known to be important for virulence regulation, host invasion, and intracellular growth control in a variety of pathogenic bacteria (Lobato-Márquez *et al.* 2016), which could indicate that the *A. adamantis* symbiont employs comparable strategies for beneficial interactions with its hosts, similar to what has been proposed for mutualistic symbionts of deep-sea mussels (Sayavedra *et al.* 2015).

### Conclusions

Using a combination of Illumina and Nanopore sequencing at an average coverage of 931×, in this study, we generated the first draft endosymbiont genome of the endemic hydrothermal vent snail *A. adamantis* from the Mariana Arc. The presented genome assembly closes a gap in the genomic resources currently available for symbionts of deep-sea provannid snails and will be useful for further analyses of host–symbiont dynamics and symbiont genome evolution according to host and environmental factors. While gene content of the *A. adamantis* symbiont appeared overall characteristic of chemosynthetic Gammaproteobacteria and related *Alviniconcha* symbionts, notable exceptions were observed, in particular, the presence of flagella-encoding loci and an abundance of genes for ammonia transport and urea usage. These differences might suggest specific adaptations to local habitat conditions at the Chamorro Seamount and possible contrasts in host–symbiont interactions relative to other gammaproteobacterial *Alviniconcha* symbioses. Future physiological and transcriptomic data paired with geochemical measurements will be helpful to address these hypotheses and determine the molecular basis underlying establishment, homeostasis, and niche adaptation of *Alviniconcha* symbioses at deep-sea hydrothermal vents.

## Supplementary Material

jkac220_Supplemental_Figure_Captions

jkac220_Supplemental_Tables

jkac220_Figure_S1

jkac220_Figure_S2

## Data Availability

Raw Illumina and Nanopore reads and the final genome assembly have been deposited in the National Center for Biotechnology Information under BioProject number PRJNA806158. The genome assembly is available under accession number JAKRWE000000000. Supplemental material is available at G3 online.
