# Supplementary material for: Genome assembly of the chemosynthetic endosymbiont of the hydrothermal vent snail Alviniconcha adamantis from the Mariana Arc
Source: G3 (Bethesda). 2022 Aug 23;12(10):jkac220. doi: 10.1093/g3journal/jkac220 (PMC9526052; doi:10.1093/g3journal/jkac220)
Supplement: jkac220_Supplemental_Figure_Captions [file jkac220_supplemental_figure_captions.docx]

**Supplementary Figure Captions**

**Fig. S1** Non-collapsed version of the phylogenetic tree shown in Fig. 2.

**Fig. S2** Functional similarity plot as shown in Fig. 3, with different *Ca.* Endoriftia genome assemblies (Table S2) labelled in black. The draft genome from Robidart *et al.* (2008), *Ca.* Endoriftia persephone RIFPA1 (AASF00000000), clusters away from the other *Ca.* Endoriftia genomes, likely due to gene annotation issues resulting from the high fragmentation of this assembly.
