# Supplementary figures and images for: Genome assembly of the chemosynthetic endosymbiont of the hydrothermal vent snail Alviniconcha adamantis from the Mariana Arc
Source: G3 (Bethesda). 2022 Aug 23;12(10):jkac220. doi: 10.1093/g3journal/jkac220 (PMC9526052; doi:10.1093/g3journal/jkac220)

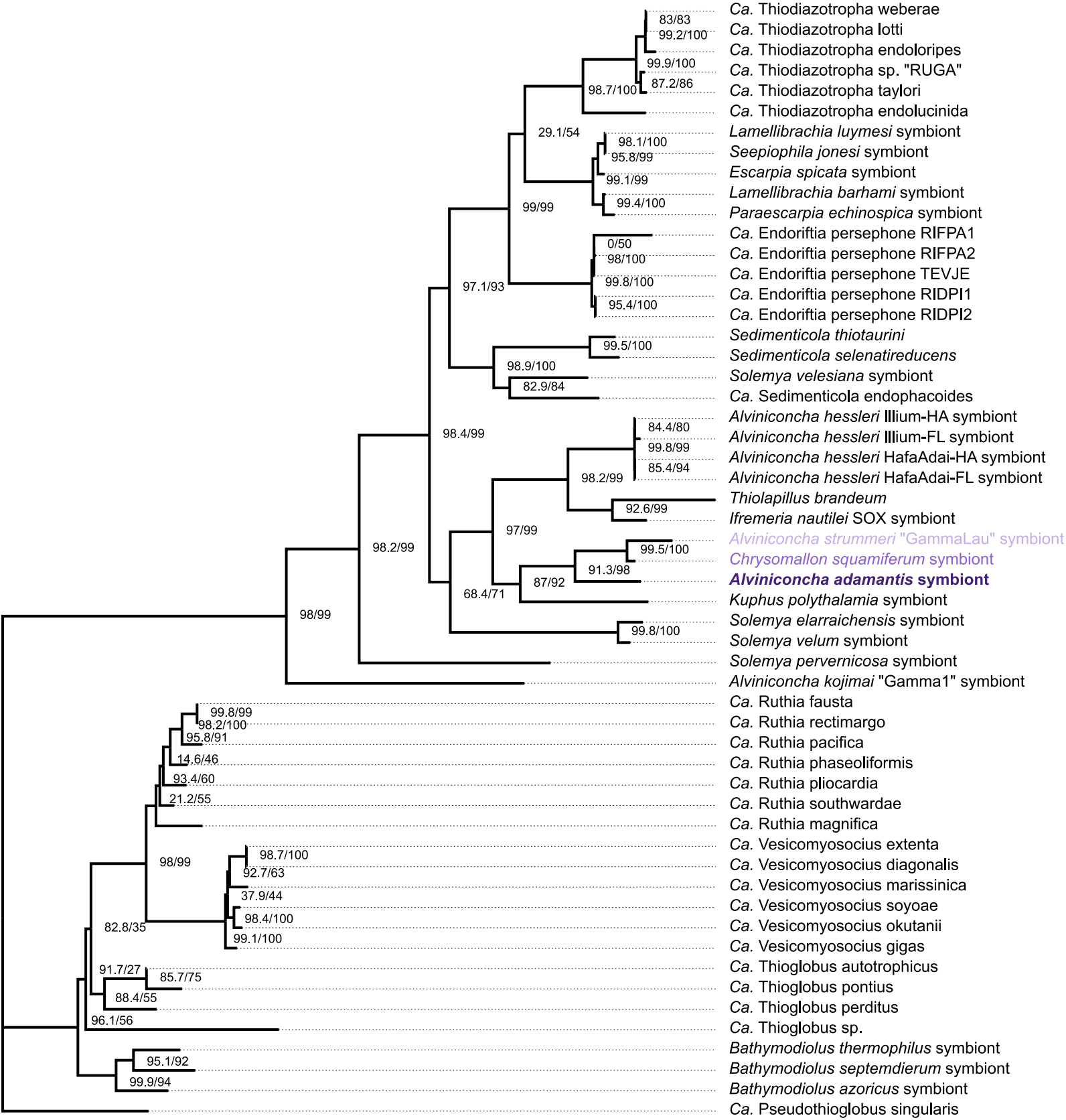

Supplement: jkac220_Figure_S1 [file jkac220_figure_s1.pdf]
