# Supplementary material for: Genome assembly of the chemosynthetic endosymbiont of the hydrothermal vent snail Alviniconcha adamantis from the Mariana Arc
Source: G3 (Bethesda). 2022 Aug 23;12(10):jkac220. doi: 10.1093/g3journal/jkac220 (PMC9526052; doi:10.1093/g3journal/jkac220)
Supplement: jkac220_Figure_S2 [file jkac220_figure_s2.pdf]

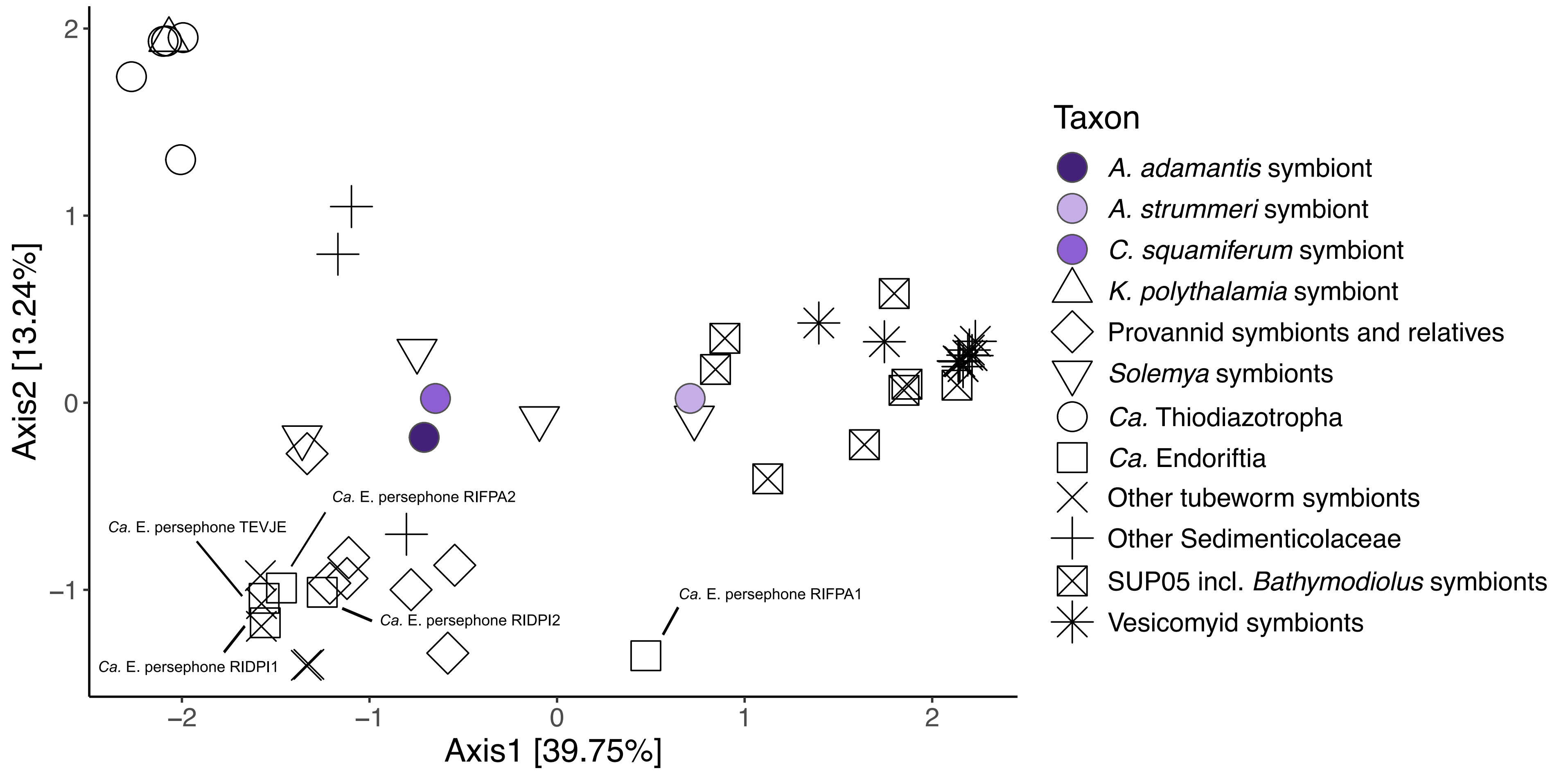

Axis2 [13.24%]
Axis1 [39.75%]
2
1
0
−1
−2
−1
0
1
2
Ca. E. persephone RIFPA2
Ca. E. persephone TEVJE
Ca. E. persephone RIDPI2
Ca. E. persephone RIDPI1
Ca. E. persephone RIFPA1
Taxon
A. adamantis symbiont
A. strummeri symbiont
C. squamiferum symbiont
K. polythalamia symbiont
Provannid symbionts and relatives
Solemya symbionts
Ca. Thiodiazotropha
Ca. Endoriftia
Other tubeworm symbionts
Other Sedimenticolaceae
SUP05 incl. Bathymodiolus symbionts
Vesicomyid symbionts
